# A copper chaperone–mimetic polytherapy for SOD1-associated amyotrophic lateral sclerosis

**DOI:** 10.1016/j.jbc.2022.101612

**Published:** 2022-01-20

**Authors:** L. McAlary, V.K. Shephard, G.S.A. Wright, J.J. Yerbury

**Affiliations:** 1 Illawarra Health and Medical Research Institute, University of Wollongong, Wollongong, New South Wales, Australia; 2 Faculty of Science, Medicine and Health, Molecular Horizons and School of Chemistry and Molecular Bioscience, University of Wollongong, Wollongong, New South Wales, Australia; 3 Department of Biochemistry & Systems Biology, Institute of Systems, Molecular and Integrative Biology, University of Liverpool, Liverpool, United Kingdom

**Keywords:** amyotrophic lateral sclerosis, protein aggregation, protein folding, superoxide dismutase 1, ebselen, CuATSM, ALS, amyotrophic lateral sclerosis, CCS, Cu chaperone for SOD1, Cu, copper, CuATSM, diacetylbis(*N*(4)-methylthiosemicarbazonato)copper(II), DMEM, Dulbecco's modified Eagle's medium-F12, DMSO, dimethyl sulfoxide, EGFP, enhanced green fluorescent protein, fALS, familial ALS, HSA, highest single agent, LED, light-emitting diode, MBR, metal-binding region, PTM, post-translational modification, SOD1, superoxide dismutase 1, TBST, TBS with Tween-20, TDP-43, TAR DNA-binding protein 43, WTL, wildtype-like, Zn, zinc

## Abstract

Amyotrophic lateral sclerosis (ALS) is a neurodegenerative disease in which motor neurons progressively and rapidly degenerate, eventually leading to death. The first protein found to contain ALS-associated mutations was copper/zinc superoxide dismutase 1 (SOD1), which is conformationally stable when it contains its metal ligands and has formed its native intramolecular disulfide. Mutations in SOD1 reduce protein folding stability *via* disruption of metal binding and/or disulfide formation, resulting in misfolding, aggregation, and ultimately cellular toxicity. A great deal of effort has focused on preventing the misfolding and aggregation of SOD1 as a potential therapy for ALS; however, the results have been mixed. Here, we utilize a small-molecule polytherapy of diacetylbis(*N*(4)-methylthiosemicarbazonato)copper(II) (CuATSM) and ebselen to mimic the metal delivery and disulfide bond promoting activity of the cellular chaperone of SOD1, the “copper chaperone for SOD1.” Using microscopy with automated image analysis, we find that polytherapy using CuATSM and ebselen is highly effective and acts in synergy to reduce inclusion formation in a cell model of SOD1 aggregation for multiple ALS-associated mutants. Polytherapy reduces mutant SOD1-associated cell death, as measured by live-cell microscopy. Measuring dismutase activity *via* zymography and immunoblotting for disulfide formation showed that polytherapy promoted more effective maturation of transfected SOD1 variants beyond either compound alone. Our data suggest that a polytherapy of CuATSM and ebselen may merit more study as an effective method of treating SOD1-associated ALS.

Over 160 mutations have been identified throughout the gene encoding copper/zinc (Cu/Zn) superoxide dismutase 1 (*SOD1*) that are known to cause the motor neuron disease amyotrophic lateral sclerosis (ALS) (1, 2). These mutations result predominantly in amino acid substitutions found in all SOD1 protein secondary structure elements, cofactor binding sites, and the homodimer interface. Each is thought to cause ALS by decreasing SOD1 folding stability, thereby creating a pool of misfolded and aggregation-prone SOD1 (3, 4). While there is debate as to whether proteinaceous aggregates or smaller and soluble non-native oligomers are toxic (5, 6, 7), the initial event considered to spark cell death is SOD1 protein misfolding (3, 4, 8, 9, 10, 11).

SOD1 maturation comprises several sequential post-translational modifications (PTMs). Initially, the spontaneous binding of Zn to immature monomer provides some folding stability (12). Zn-bound SOD1 then associates with the Cu chaperone for SOD1 (CCS), which facilitates the input of Cu and subsequent oxidation of an intrasubunit disulfide bond between Cys57 and Cys146. The stable monomer is then free to form enzymatically active homodimers (13, 14, 15). Metal-binding region (MBR) mutants affect Cu or Zn coordination and activity, whereas wildtype-like (WTL) mutants retain high levels of enzymatic activity when mature (16, 17). Immature SOD1, lacking PTMs, is prone to misfolding and is the central component of intracellular aggregates found within SOD1-associated ALS neuronal tissues (3, 8, 10, 11, 18, 19, 20), whereas mature SOD1 is highly stable (21). Maturation and misfolding are therefore antagonistic pathways that dictate SOD1 toxicity. A cell has finite resources and a limited capacity to catalyze nascent SOD1 maturation. The maturation pathway can be overwhelmed by a high concentration of nascent SOD1 (22) or inhibited by mutations that prevent cofactor binding and disulfide PTMs (3, 4, 9, 23, 24). This results in increased misfolding pathway flux and the proteostasis pathways becoming overwhelmed (25). Decreasing SOD1 expression and thereby reducing traffic along the misfolding pathway is the focus of knockdown strategies currently in clinical trials (26). However, SOD1 has important metabolic functions, and long-term ablation of its activity is known to have detrimental effects. Another therapeutic option is to increase maturation or cellular proteostasis capacity. Heat shock protein molecular chaperone upregulation reduces SOD1 misfolding, and clinical trials are ongoing (27). In addition, several small molecules have been shown to act as direct pharmacological chaperones for SOD1 (28, 29, 30, 31). Pyrimidine derivatives, 5-fluorouridine and telbivudine, reduce SOD1 *in vitro* aggregation and *in vivo* toxicity, respectively (28, 29). Treatment of SOD1-G93A mice with the 5-fluorouridine analog, 5-fluorouracil, also delays symptom onset and increases survival (32).

The cognate chaperone of SOD1, CCS, is a uniquely placed chaperone that has evolved to increase SOD1 maturation pathway throughput. It exerts molecular, Cu, and oxidative folding chaperone activity on nascent SOD1 mediated through a specific protein–protein interaction (13, 14, 33, 34). While overexpression of human CCS reduces the accumulation of misfolded SOD1, it can also recruit SOD1 mutants to the mitochondrial intermembrane space where they accelerate vacuolization and toxicity (35, 36, 37). Two small molecules have been shown to recapitulate CCS activities. Diacetylbis(*N*(4)-methylthiosemicarbazonato)copper(II) (CuATSM) promotes WTL mutant SOD1 Cu binding in several mouse models and increases life span (31, 38, 39, 40), but it is ineffective against MBR mutants (41). The seleno-organic compound ebselen promotes the formation of the SOD1 intrasubunit disulfide bond in cultured cells (30) and increases SOD1 dimer affinity (42, 43). As there are few SOD1 ALS-associated mutations that directly prevent disulfide formation (C146R and truncation mutants), ebselen is likely to be effective for MBR mutants as well as WTL (2). Indeed, recent evidence shows that ebselen and some of its derivatives can restore the viability of cultured cells expressing G93A mutant SOD1 as well as delay disease onset in G93A mice through dietary supplementation (44).

Here, we report the effect of ebselen on intracellular mutant SOD1 inclusion formation in a disease-relevant cell model. To achieve this, we developed a machine learning–based image analysis pipeline for accurate measurement of protein inclusion formation in large microscopy datasets. Application of this method to a subset of compounds showed ebselen was capable of reducing inclusion formation for both WTL and MBR SOD1 mutants. We then utilized this method to investigate CuATSM and ebselen cotherapy aiming to divert nascent SOD1 from the misfolding pathway to the maturation pathway and thereby reduce mutant toxicity. We show that these compounds can act in a synergistic manner to reduce SOD1 aggregation through the promotion of dimerization, disulfide formation, and promoting increased levels of active SOD1. All mutants analyzed display positive outcomes for at least one marker of effective SOD1 maturation with resulting cell viability increases for common or severely structurally destabilizing mutants. This work highlights the unexplored possibilities of mutation-specific personalized therapy for SOD1–ALS and the potential use of a CCS-mimetic polytherapy specifically targeting steps on the SOD1 PTM maturation pathway.

## Results

### Automated image analysis to identify cells containing inclusions

We, and others, have previously utilized genetically encoded fluorescent proteins as a tool to investigate the inclusion formation of SOD1 in cultured cells finding that ALS-associated and *de novo* mutations, as well as small molecules, can alter this process (3, 29, 41, 45, 46, 47, 48). We have also utilized several methods to detect fluorescent proteinaceous inclusions, including manual counting (3), fluorescence intensity thresholding (29), and cell permeabilization to release soluble GFP-tagged protein (29, 41, 49). We now sought to enhance our detection of protein inclusion formation in cells for use in larger mutational or drug screens. This was accomplished by exploiting advances made in the area of microscopy image analysis with the application of user-assisted machine learning to accurately classify cellular phenotypes (50).

**Figure 1:**
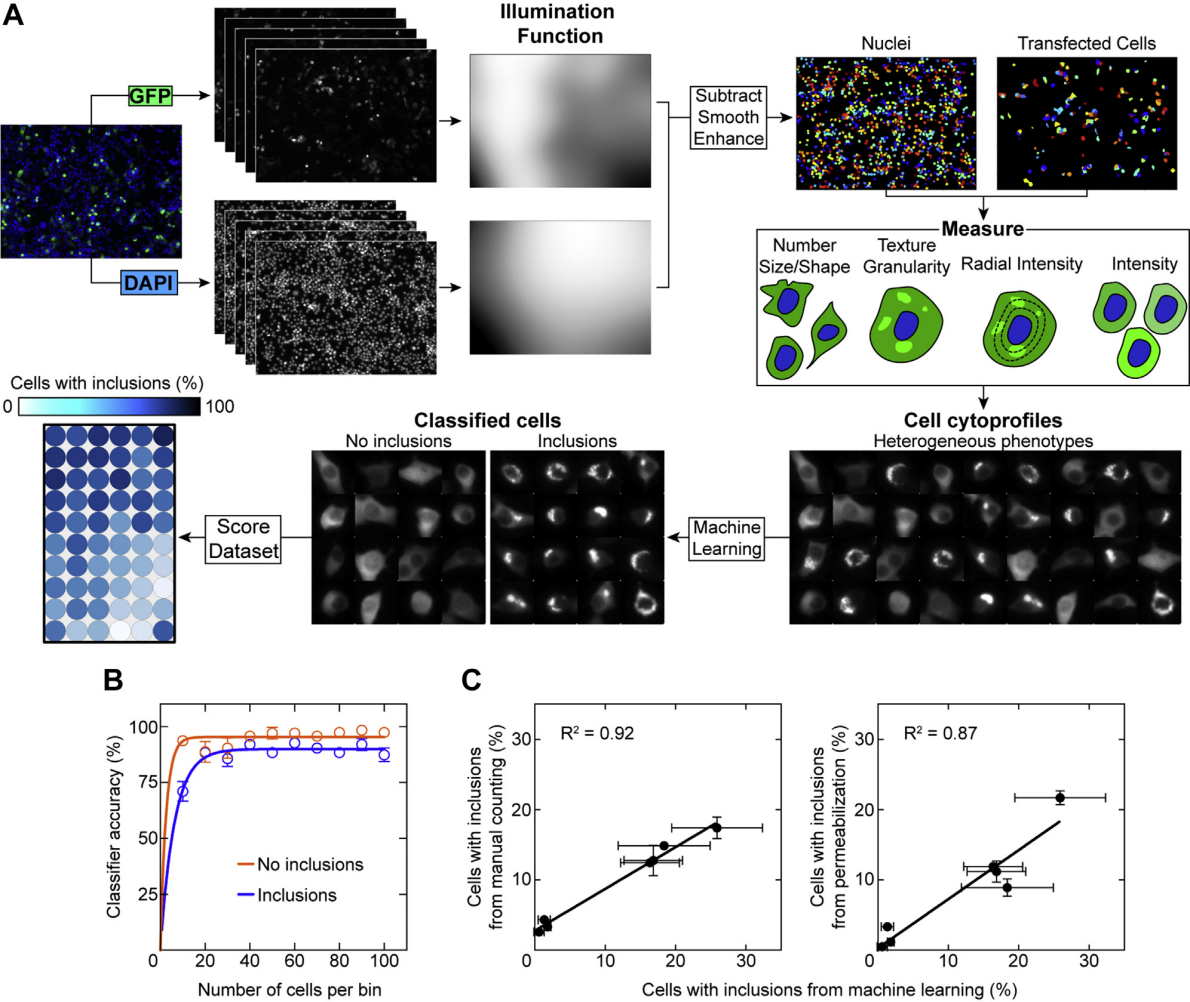
**User-assisted machine learning to determine cells containing inclusions.***A*, the image analysis pipeline first performs illumination correction for both DAPI and GFP channels and then segments the transfected cells for measurement. A user then identifies phenotypes in a small subset of the cell population to train the classifier for identification of the entire cell population. *B*, the classifier requires less than 100 to become accurate at categorizing cells into inclusion containing (*blue*) and those that did not contain inclusions (*orange*). *C*, correlations of the percentage of cells with inclusions in this work *versus* previously published examination of the same SOD1-EGFP expression constructs in NSC-34s (*left*) *versus* manual counts from McAlary *et al.*, 2016 (3) and (*right*) *versus* saponin-permeabilized cells from Farrawell *et al.* (25) 2018. Error bars represent SD of the mean from at least three separate classification requests. DAPI, 4′,6-diamidino-2-phenylindole; EGFP, enhanced GFP; SOD1, superoxide dismutase 1.

**Supplement Figure S1:**
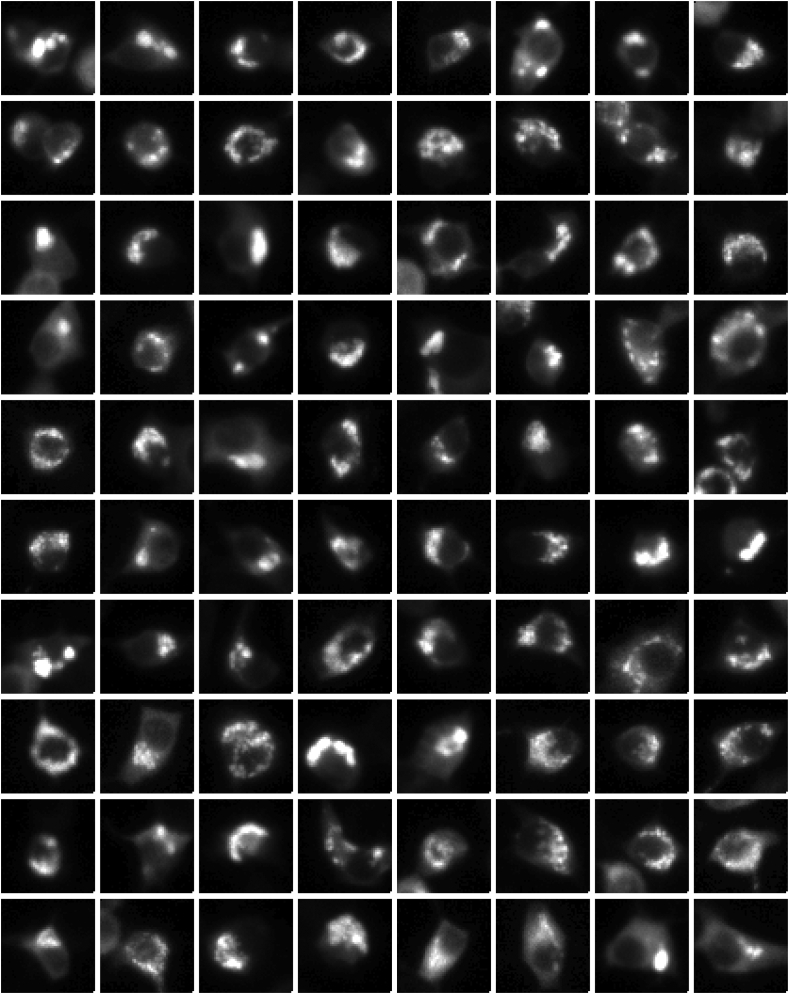
Example thumbnails of cells containing inclusions from the entire set of data used for this work, showing the heterogeneity of inclusion formation.

**Supplement Figure S2:**
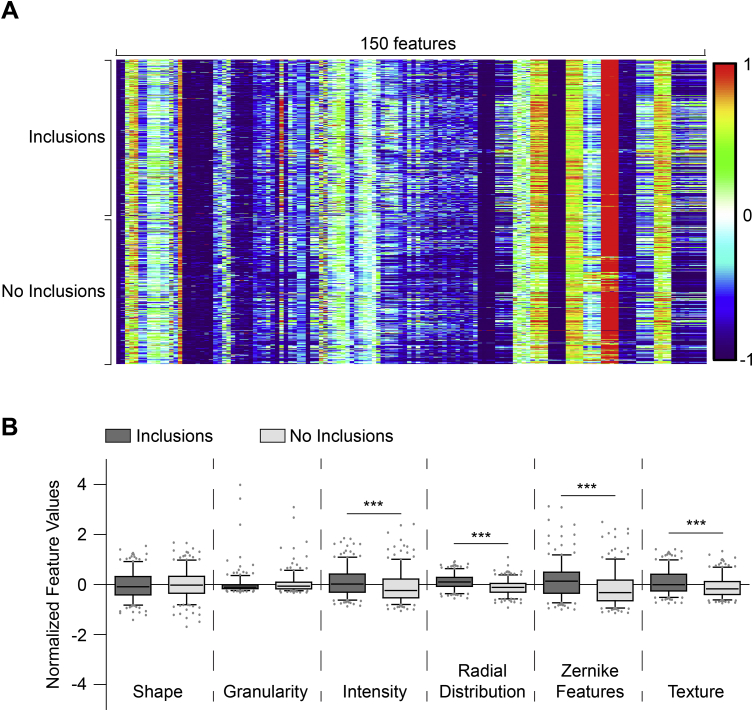
Key measurements used to generate the cytoprofiles of cells. *A*, heat map of the 150 features that were measured from 150 cells in each classification group (Inclusions *versus* No Inclusions). *B*, Z-normalised values from each extracted feature for both cells with inclusions (*dark gray*) and cells without inclusions (*light gray*). Data are plotted as box-whisker plots where whiskers show the 5 to 95% range, the box the 25 to 75% range and the middle bar the median values. Significance was determined using student’s t-test (∗∗∗*p* ≤ 0.001).

To this end, we developed an image analysis pipeline using CellProfiler software (Broad Institute, MIT Harvard) (51) to identify and segment transfected cells for measurement followed by classification in CellProfiler Analyst software (Fig. S1). Measurement parameters were chosen to append spatial data (shapes, texture, granularity, radial intensity, and intensity) in order to generate cytoprofiles that were processed by a random forest classifier (Figs. 1*A* and S2*A*) (50). Normalization of the extracted cellular features demonstrated significant differences in texture, radial intensity, and intensity measurements but not shape or granularity (Fig. S2*B*). This indicated that these measurements were appropriate for the profiling of cells. We found that our segmentation parameters correctly identified transfected cells with high accuracy (97 ± 2%) and that the chosen measurements effectively facilitated accurate classification for both cells with and without inclusions (95 ± 0.5% and 98 ± 0.9% accuracy, respectively) (Fig. 1*B*).

### Ebselen, but not other compounds, reduces the formation of ALS-associated mutant SOD1 inclusions in cultured cells

Having established a rapid and accurate image-based method of classifying cells containing inclusions, we next sought to examine the effect of ebselen and a small panel of other similar small molecules on SOD1 inclusion formation. The molecules, other than ebselen, were omeprazole, clopidogrel, and lipoic acid. These compounds were chosen on the basis that they contained sulfur moieties that may be redox active in a similar manner to ebselen (30).

**Figure 2:**
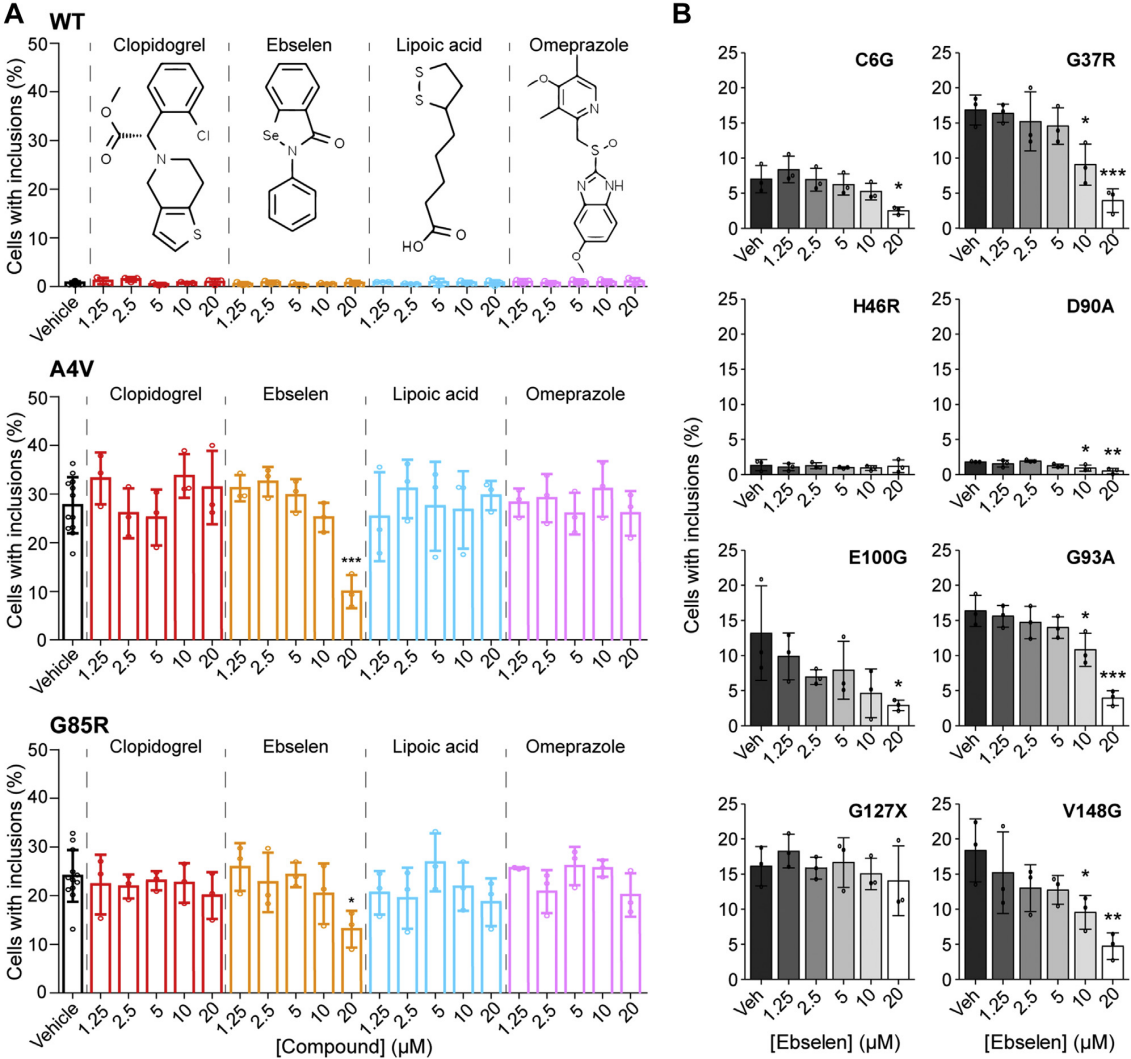
**Ebselen reduces inclusion formation of SOD1 ALS-associated mutants in cultured cells.** NSC-34 cells expressing (*A*) SOD1-EGFP variants SOD1^WT^, SOD1^A4V^, and SOD1^G85R^ were treated with vehicle (*black*), clopidogrel (*red*), ebselen (*orange*), lipoic acid (*teal*), or omeprazole (*pink*) for 48 h, and the number of cells containing inclusions was enumerated. *B*, NSC-34 cells expressing SOD1-EGFP variants SOD1^C6G^, SOD1^G37R^, SOD1^H46R^, SOD1^D90A^, SOD1^E100G^, SOD1^G93A^, SOD1^G127X^, and SOD1^V148G^ were all treated with increasing concentrations of ebselen, and inclusion formation was measured. Ebselen treatment decreased inclusion formation for most variants except for SOD1^G127X^ and SOD1^H46R^. Error bars represent SD of the mean of at least three separate experiments. Statistical significance was determined using Student's *t* test against vehicle control (∗∗∗*p* < 0.001; ∗*p* < 0.05). ALS, amyotrophic lateral sclerosis; EGFP, enhanced GFP; SOD1, superoxide dismutase 1.

**Supplement Figure S3:**
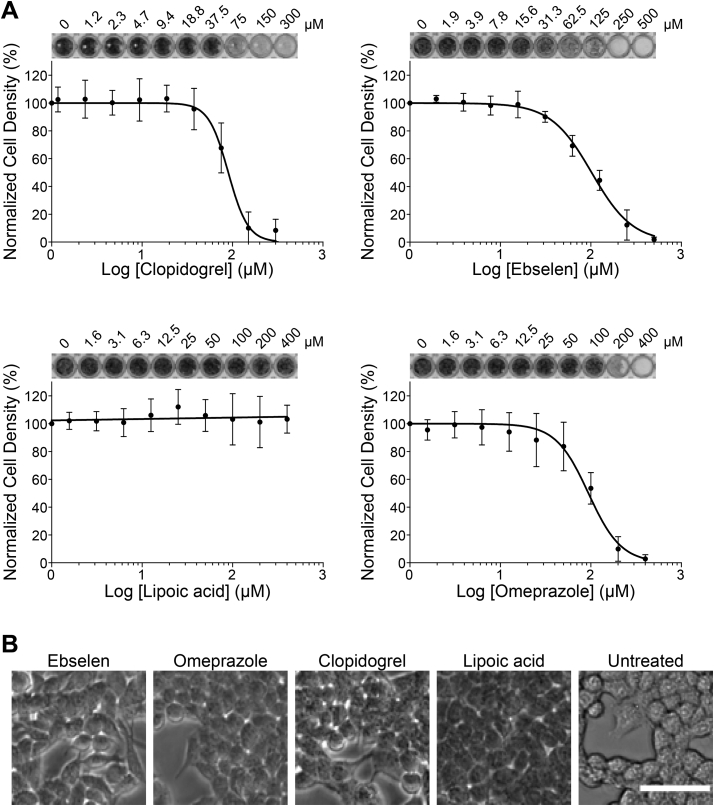
Determination of the non-toxic concentration of compounds to treat NSC-34 cells. *A*, crystal violet assay determined NSC-34 densities following treatment with either clopidogrel, ebselen, lipoic acid, or omeprazole. Above each plot is an example set of well images showing the crystal violet staining of cells at different compound concentrations. *B*, phase contrast imaging of NSC-34 cells at a concentration of 20 μM for each drug. Scale bar = 100 μm. Error bars represent SD of the mean from at least 3 separate biological replicates.

To this end, NSC-34 cells were transfected with SOD1 variants SOD1^WT^, SOD1^A4V^ (WTL mutant), or SOD1^G85R^ (MBR mutant) and were treated with determined nontoxic concentrations of ebselen, lipoic acid, omeprazole, or clopidogrel (Fig. S3) for 48 h prior to being fixed, imaged, and analyzed. We observed that SOD1^WT^ formed very few inclusions across treatments, with only 0.8 ± 0.3% of cells in the untreated group being classified as containing inclusions (Fig. 1*A*), in line with previous observations by us and others (3, 29, 41, 45, 46, 47, 48). In contrast, both SOD1^A4V^ and SOD1^G85R^ readily formed inclusions in this system with 27.7 ± 5.8% and 24.1 ± 5.3% cells containing inclusions, respectively (Fig. 2*A*). Treatment of SOD1^A4V^ or SOD1^G85R^-expressing cells with either lipoic acid, omeprazole, or clopidogrel at any of the tested concentrations had no significant effect on inclusion formation (Fig. 2*A*). We found that treatment with ebselen at the highest concentration of 20 μM resulted in a significant reduction in both SOD1^A4V^ and SOD1^G85R^ inclusion formation (Fig. 2*A*), suggesting that ebselen was protective against both WTL and MBR mutant inclusion formation in this model. The reduction in SOD1^A4V^ mutant inclusion formation was more substantial (threefold decrease) when compared with SOD1^G85R^ (twofold decrease). Previous examination of the capability of ebselen to facilitate SOD1 maturation in cells used a 10-fold greater concentration of ebselen (200 μM) than we used here (30), where our results suggest that ebselen may be more potent at facilitating SOD1 maturation than previously suggested.

Ebselen was further tested on SOD1 variants including SOD1^C6G^, SOD1^G37R^, SOD1^H46R^, SOD1^D90A^, SOD1^G93A^, SOD1^E100G^, SOD1^G127X^, and SOD1^V148G^ in this model (see Table S1 for mutant properties). Most of these ALS-associated mutants in this list are WTL and induce inclusion formation to various degrees in NSC-34 cells (3, 41). SOD1^H46R^ and SOD1^G85R^ are MBR mutants with minimal ability to bind Cu (52, 53), and SOD1^G127X^ is a truncation mutant that removes residues 127 to 153, including the disulfide forming Cys146 residue (54). Treatment of the transfected NSC-34 cells with increasing concentrations of ebselen showed that for cells expressing SOD1^C6G^, SOD1^G37R^, SOD1^D90A^, SOD1^G93A^, SOD1^E100G^, and SOD1^V148G^, there was a significant dose-dependent response to ebselen (Fig. 2*B*). The most effective dose observed in each case was 20 μM, although a significant difference between vehicle control and a concentration of 10 μM was observed for SOD1^G37R^, SOD1^D90A^, SOD1^G93A^, and SOD1^V148G^ (Fig. 2*B*), indicating a greater effective action of ebselen on these mutants. Cells expressing the truncated SOD1^G127X^ mutant, which does not contain the disulfide-forming Cys146 residue, showed no significant effect of ebselen on the percentage of cells containing inclusions at any concentration tested (Fig. 2*B*). SOD1^H46R^ transfected cells showed no response to ebselen; however, inclusion formation is low for this mutant. These data indicate that ebselen is likely acting through the previously proposed method of promoting the formation of the SOD1 intrasubunit disulfide between residues Cys57 and Cys146 (30).

### A combination of ebselen and CuATSM is effective at decreasing SOD1 inclusion formation in cells

Multidrug polytherapies are used as a standard treatment against most types of cancer and other diseases (55). This is a less common but growing strategy being adopted against neurodegenerative diseases (56, 57). Previous research has shown that CuATSM is capable of facilitating Cu delivery to neuronal SOD1 in animals and cells (31, 41). Likewise, we have shown that ebselen can promote SOD1 disulfide formation in living cells (30). Therefore, we reason that CuATSM and ebselen have the potential to act collaboratively to promote both Cu binding and disulfide formation, respectively, in SOD1 mutants (30, 31, 41).

**Supplement Figure S4:**
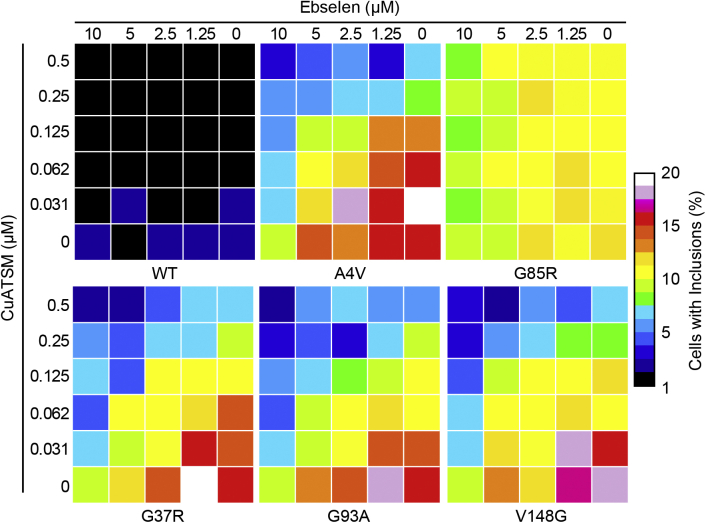
Heat map of SOD1-EGFP variants inclusion formation in a checkerboard assay of CuATSM and ebselen. Related to main text Figure 3.

Considering this, we set out to establish if a combination of both CuATSM and ebselen would have a greater effect at reducing SOD1-associated familial ALS (fALS) phenotypes in our cell model than either drug alone. When investigating the effects of two drugs given in combination, analyses take into account the biological effect of the drugs to determine correct synergy models to apply (58). In our case, the highest single agent (HSA) model is the most appropriate considering that both ebselen and CuATSM act in a similar manner by promoting correct SOD1 folding through acquisiton of PTMs. To this end, we performed a checkerboard treatment of NSC-34 cells expressing SOD1^WT^, SOD1^A4V^, SOD1^G37R^, SOD1^G85R^, SOD1^G93A^, or SOD1^V148G^ using different concentrations of CuATSM (0.5–0 μM) with ebselen (10–0 μM). SOD1^WT^ formed very few inclusions in this assay at all CuATSM–ebselen combination treatments (Fig. S4). In comparison, all ALS-associated variants formed more inclusions at lower drug concentrations (Fig. S4). Heatmap visualization of the inclusion formation shows that the WTL mutants (SOD1^A4V^, SOD1^G37R^, SOD1^G93A^, and SOD1^V148G^) responded well to both CuATSM and ebselen monotherapy, whereas SOD1^G85R^ shows no response to CuATSM monotherapy but responds to higher concentrations of ebselen monotherapy (Fig. S4).

**Figure 3:**
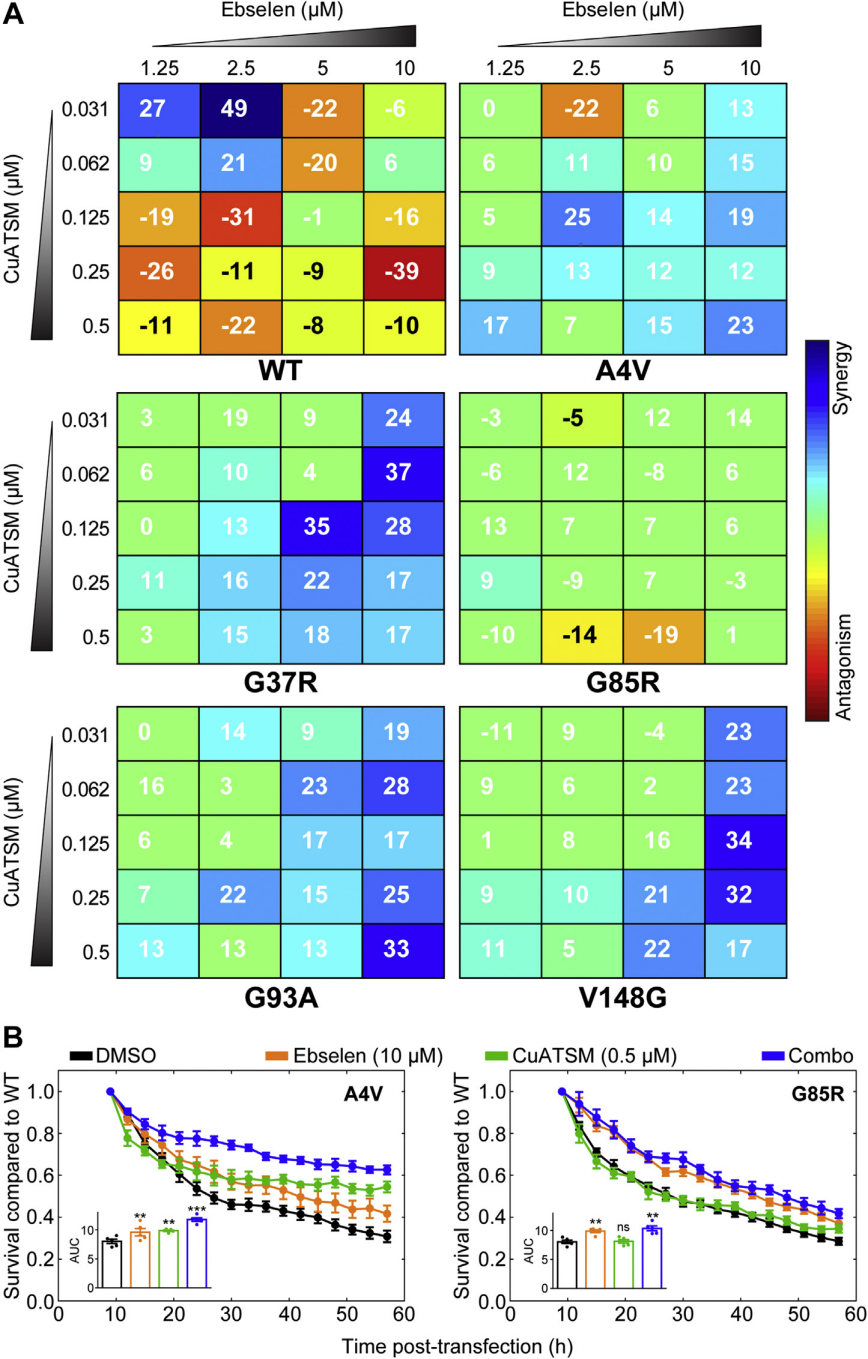
**CuATSM and ebselen polytherapy is more effective than monotherapy at reducing WT-like SOD1 mutant inclusion formation.***A*, highest single agent (HSA) heatmaps of CuATSM and ebselen checkerboard treatment of NSC-34 cells expressing either SOD1^WT^ (*top left*), SOD1^A^^4V^ (*top right*), SOD1^G^^37R^ (*middle left*), SOD1^G^^85R^ (*middle right*), SOD1^G^^93A^ (*bottom left*), and SOD1^V^^148G^ (*bottom right*). Color scale represents the degree to which antagonism or synergy is occurring. Numbers in *boxes* represent mean values from three separate experiments, and a number above 10 is considered as synergistic at those concentrations. *B*, transfected cell counts of NSC-34 cells expressing SOD1^A^^4V^ (*left*) and SOD1^G^^85R^ (*right*) treated with vehicle DMSO (*black*), ebselen (10 μM; *orange*), CuATSM (0.5 μM; *green*), and ebselen/CuATSM combo (10 μM/0.5 μM; *blue*). Cell counts are relative to SOD1^WT^ transfected cells treated with the same compounds. *Inset* in each panel is the area under the curve measurements for each drug treatment. Error bars represent SEM of three separate experiments. Statistical significance was determined using a one-way ANOVA with Dunnet's test against DMSO-treated cells (∗∗∗*p* < 0.01; ∗∗*p* < 0.05). CuATSM, diacetylbis(*N*(4)-methylthiosemicarbazonato)copper(II); DMSO, dimethyl sulfoxide; SOD1, superoxide dismutase 1.

Using the Combenefit software (Cancer Research UK Cambridge Institute) (59), we analyzed our reduction in inclusion formation for HSA synergy. Ebselen and CuATSM acted synergistically for all WTL mutants but not SOD1^G^^85R^ or SOD1^WT^ (Fig. 3*A*). Generally, there was a trend toward higher concentrations of both ebselen and CuATSM acting in a more synergistic manner. Both drugs were capable of reducing the concentration of the other drug needed to obtain a reduction in inclusions. Indeed, ebselen at a concentration of 10 μM was capable of significantly reducing the number of inclusions in cells expressing the aggressive SOD1^A^^4V^ mutant to roughly 5% with as little as 0.03 μM CuATSM, which is over a ten fold decrease in the maximum CuATSM concentration (Fig. S4). Likewise, for SOD1^A^^4V^ again, a concentration of 0.25 μM CuATSM was capable of reducing the necessary ebselen concentration for significant inclusion formation reduction by roughly eightfold, from 10 to 1.25 μM. These effects were also observed for the other WTL mutants examined in this assay (Fig. S4).

We next performed a live-cell time-lapse microscopy assay to count the relative numbers of GFP-positive cells across time under the most potent treatment regimes used in the checkerboard assay (CuATSM at 0.5 μM and ebselen at 10 μM). Data are reported as the number of cells that are enhanced GFP (EGFP)-positive relative to SOD^WT^–EGFP transfected cells treated with CuATSM, or ebselen, or a combination of both compounds. The expectation is that the compounds would reduce the time-dependent decline in relative to GFP-positive cell numbers. Similar to previous reports (3, 25, 29, 41), transfection of NSC-34 cells with mutant SOD1-EGFP results in a decline in relative EGFP-positive cells over time (Fig. 3*B*; *left* SOD1^A4V^, *right* SOD1^G85R^). In this assay, cells transfected with SOD1^A4V^ and treated with either ebselen or CuATSM alone or with the combination therapy saw a significant increase in the number of GFP-positive cells across time as determined by measuring the area under the curve (Fig. 3*B*; *left* and *inset*). Cells transfected with SOD1^G85R^ also showed a similar trend of decreasing numbers of relative GFP-positive cells across time; however, CuATSM treatment alone had no effect on this decline, whereas ebselen alone and ebselen with CuATSM in combination did significantly reduce the decline in cell numbers relative to SOD1^WT^ (Fig. 3*B*; *right* and *inset*).

### Polytherapy with ebselen and CuATSM mimics CCS activity and is effective at promoting mutant SOD1 maturation

**Figure 4:**
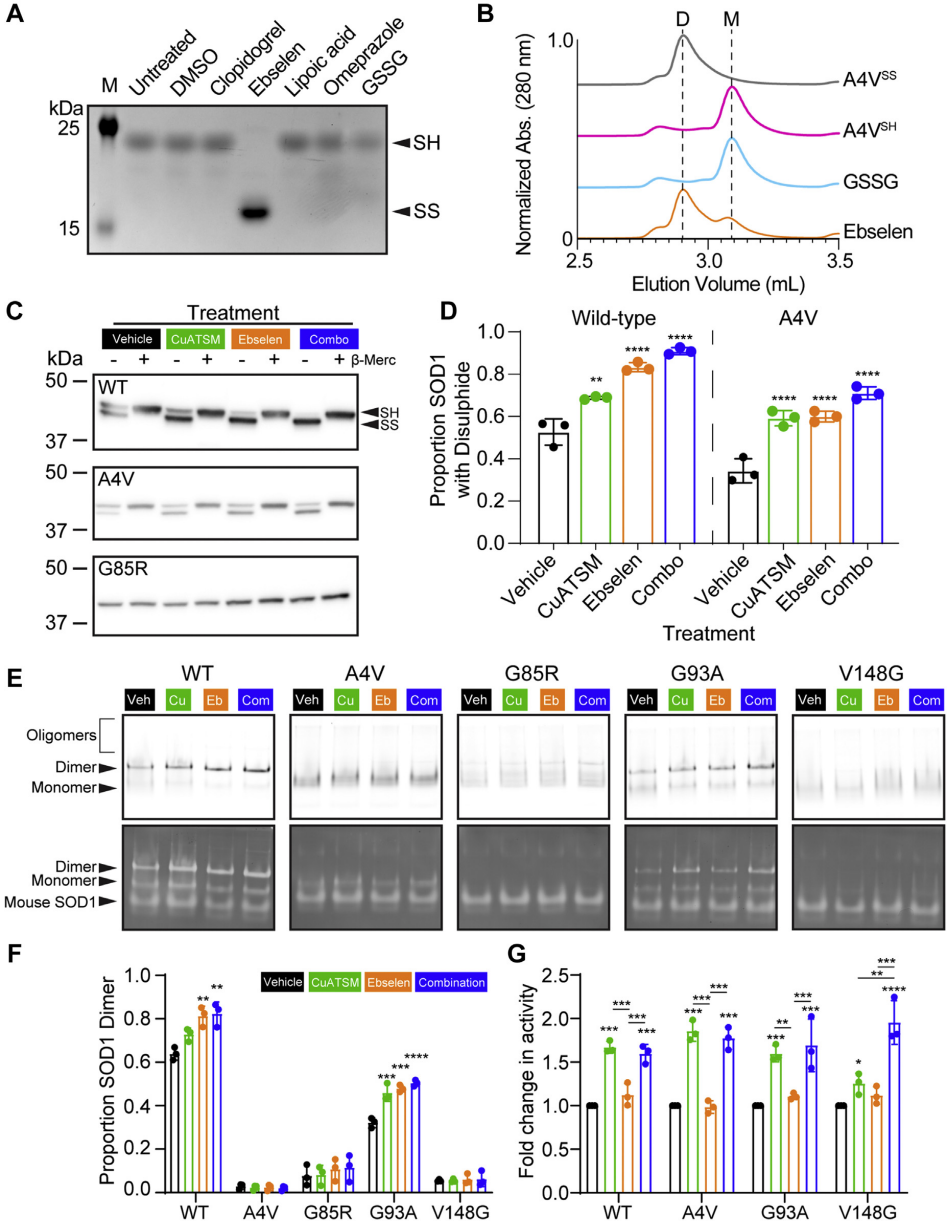
**A combination treatment of CuATSM and ebselen is effective at rescuing SOD1 ALS-associated mutant folding.***A*, nonreducing SDS-PAGE AMS assay shows that treatment with ebselen but not other redox-containing compounds resulted in recombinant SOD1^A^^4V^ disulfide formation (reduced = SH, oxidized intact = SS). *B*, all size-exclusion chromatographies show that ebselen promotes recombinant SOD1^A^^4V^ homodimerization, whereas oxidized glutathione (GSSG) does not (D = dimers, M = monomers). *C*, differential SDS-PAGE migration of SOD1-EGFP from cell lysates under reducing (+β-merc) and nonreducing (−β-merc) conditions shows that the proportion of disulfide-bonded SOD1 (SS) is increased with CuATSM (*green*; 0.5 μM), ebselen (*orange*; 20 μM), and a combination treatment (*blue*; 0.5 μM CuATSM/20 μM ebselen) compared with vehicle control (*black*) for both SOD1^WT^ and SOD1^A4V^, but SOD1^G85R^ remains fully reduced. *D*, densitometry of disulfide formation immunoblots for SOD1^WT^ and SOD1^A4V^ showed that CuATSM, ebselen, and the combination therapy were capable of promoting disulfide formation in living cells. *E*, native-PAGE of SOD1–TdTomato lysates shows oligomers, dimers, and monomers of SOD1 variants (*top*) and in-gel zymography of the same gels shows the relative activity of each species, including dimer, monomer, and mouse SOD1. *F*, quantification of the fluorescence signal from native-PAGE of the proportion of SOD1–TdTomato signal present for the dimer, showing that CuATSM, ebselen, or combination therapy promoted the dimerization of both SOD1^WT^ and SOD1^G93A^, but not SOD1^A4V^, SOD1^G85R^, and SOD1^V148G^. *G*, quantification of the achromatic bands from in-gel zymography, normalized to TdTomato fluorescence signal, showing that only treatment with CuATSM and the combination therapy increased the relative levels of active SOD1 for SOD1^WT^, SOD1^A4V^, SOD1^G93A^, and SOD1^V148G^ (SOD1^G85R^ not shown because of lack of activity). Error bars represent SD of the mean of at least three separate experiments. Significance was determined using one-way ANOVA with Tukey’s multiple comparisons test (∗∗∗*p* < 0.001, ∗∗*p* < 0.01, ∗*p* < 0.05). ALS, amyotrophic lateral sclerosis; AMS, acetamido-4′-maleimidylstilbene-2,2′-disulfonic acid; CuATSM, diacetylbis(*N*(4)-methylthiosemicarbazonato)copper(II); EGFP, enhanced GFP; SOD1, superoxide dismutase 1.

To investigate the mechanisms by which ebselen and CuATSM catalyze SOD1 maturation, we assessed the intrasubunit disulfide bond formation, dimerization, and activity of SOD1. In comparison to the other redox compounds examined previously, only ebselen was able to facilitate purified recombinant SOD1^A^^4V^ disulfide formation (Fig. 4*A*). Intrasubunit disulfide formation is known to shift the SOD1 monomer–dimer equilibrium in favor of the dimer (60) and stabilizes the disulphide subloop, thereby limiting solvent access to the Cys57–C146 bond. We have previously shown that ebselen binding to Cys111 can increase recombinant SOD1^A^^4V^ homodimer affinity. However, this effect is negated by the presence of dithiothreitol or reduced glutathione at physiological concentrations of 1 mM (30). Ebselen, unlike oxidized glutathione, was able to facilitate the formation of recombinant SOD1^A^^4V^ homodimers even in the presence of 5 mM reduced glutathione (Fig. 4*B*). Under these conditions, ebselen cannot form stable conjugates at Cys111; therefore, SOD1^A^^4V^ homodimerization likely results from the catalyzed formation of the SOD1 intrasubunit disulfide formation as we previously described in live cells (30).

**Supplement Figure S5:**
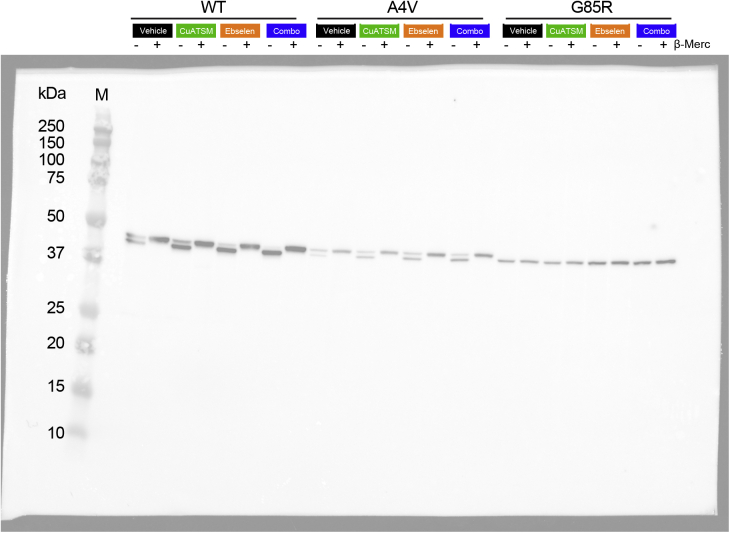
Full immunoblot representation of the electrophoretic migration assay to determine SOD1 disulfide status using SOD1-EGFP constructs. Related to main text Figure 4 panels *C* and *D*.

Next, we compared the effectiveness of CuATSM and ebselen monotherapies and combination therapy on their ability to promote the folding of transgenic intracellular SOD1 variants. Previous work determined that nonreducing SDS-PAGE of SOD1 maintains the intramolecular disulfide bond and that the disulfide bonded form of monomeric SOD1 migrates more rapidly during electrophoresis (14). SOD1 containing the intrasubunit disulfide was detected *via* immunoblot across treatment groups for both SOD1^WT^ and SOD1^A4V^ but not for SOD1^G85R^ (Figs. 4*C* and S5). Semiquantitative measurement of the immunoblots showed a significant increase in the proportion of disulfide-containing SOD1 detected for both SOD1^WT^ and SOD1^A4V^ for each treatment in comparison to vehicle control (Fig. 4*D*). Out of the treatments, the combination treatment showed the greatest increase in the proportion of SOD1 containing the intrasubunit disulfide for both SOD1^WT^ and SOD1^A4V^ (Fig. 4, *C* and *D*). The lack of detection of disulfide-bonded SOD1^G85R^ may be a result of this mutant being highly destabilized even in cells and being highly susceptible to reduction even when free thiols are chemically blocked (61, 62, 63).

**Supplement Figure S6:**
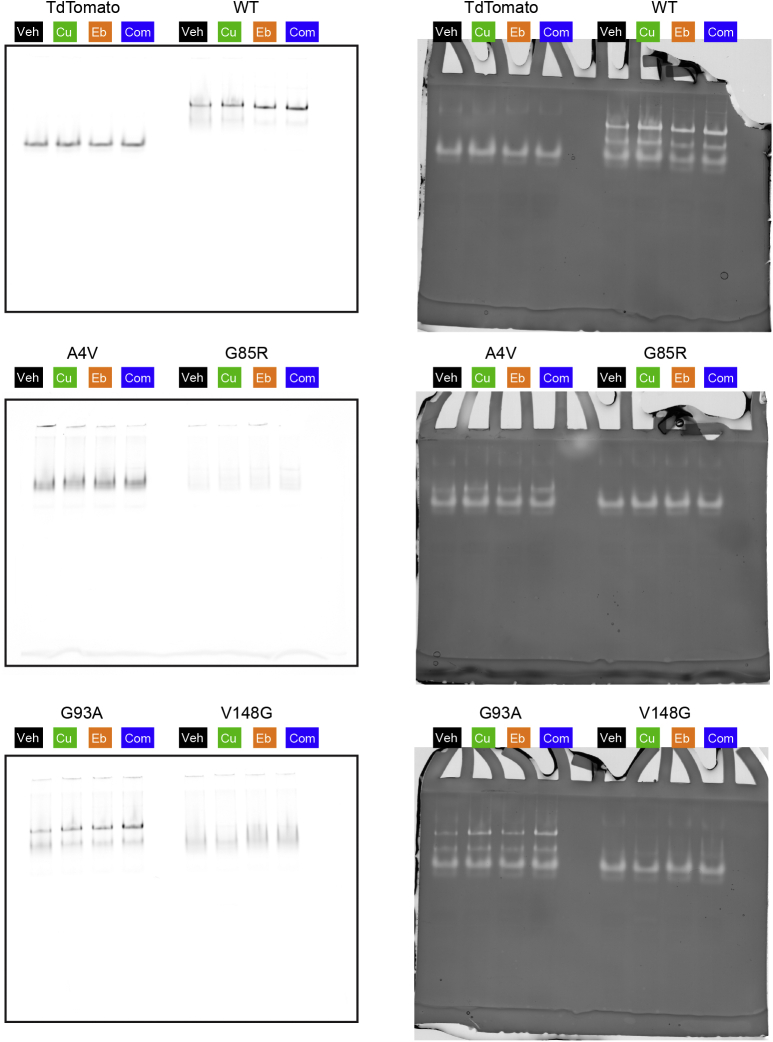
Full Native-PAGE images from TdTomato fluorescence (*left*) and in-gel zymography (*right*). Related to main text Figure 4 panels *E* and *F*.

**Supplement Figure S7:**
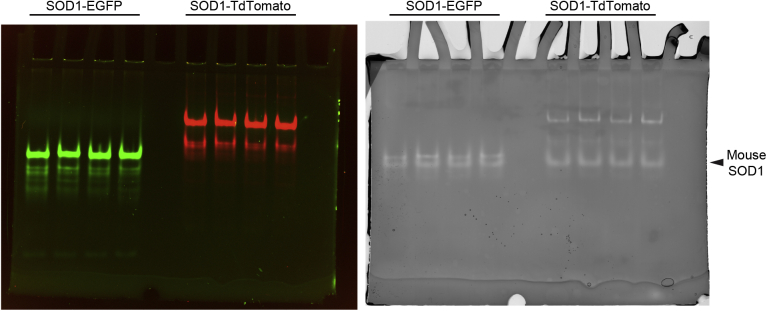
EGFP-tagged SOD1 migrates closely to mouse SOD1 in Native-gel electrophoresis. Note overexposure is to show monomer species.

We next examined the effect of CuATSM and ebselen monotherapies and polytherapy on SOD1 dimerization and activity in cells by using native-PAGE (Fig. S6) and in-gel zymography (64). Here, we used SOD1 variants tagged with TdTomato because of EGFP-tagged SOD1 migrating too close to endogenous cellular SOD1 for accurate densitometry measurements (Fig. S7). Assessment of the SOD1–TdTomato signal in native-PAGE showed that dimer was only the most prominent species for SOD1^WT^ (60% of total TdTomato signal) when cells were treated with vehicle control. Other variants were predominantly monomeric, where SOD1^G93A^ was the ALS-associated variant with the highest proportion of dimer (Fig. 4, *E* and *F*). Treatment with CuATSM, ebselen, or combination therapy showed increases in the proportion of dimeric SOD1 for both SOD1^WT^ and SOD1^G93A^, indicating that compounds were promoting dimerization either through Cu input or disulfide formation (Fig. 4, *E* and *F*). Subsequent in-gel zymography was performed, where achromatic bands indicating SOD1 activity were normalized to corresponding TdTomato fluorescent signal. This analysis showed that all variants (SOD1^G85R^ data not shown because of no activity observed under any treatment) exhibited a significant increase in the activity of SOD1–TdTomato for CuATSM and combination therapy–treated cells compared with vehicle-treated cells (Fig. 4, *E* and *G*). Ebselen treatment did not appear to result in greater levels of SOD1 activity, supporting a mechanism of stabilization that is related only to disulfide formation (30). SOD1^V148G^ was more enzymatically active when both CuATSM and ebselen were used in combination, as compared with when CuATSM was administered alone, perhaps because of ebselen further stabilizing the disulfide bond, which is an important PTM for SOD1 activity (65). Together, these results support that ebselen and CuATSM contribute to the formation of stable SOD1 molecules in living cells.

## Discussion

Considering that ALS-associated mutations in SOD1 disrupt its maturation, some therapeutic strategies have focused on catalyzing proper SOD1 folding (30, 41, 66, 67, 68). Initial methods to stabilize SOD1 were focused on promoting the formation or maintenance of the SOD1 homodimer (66, 69), which was a strategy based on the success of small molecules that maintained familial amyloid polyneuropathy–associated mutants of the serum protein transthyretin in its native tetrameric conformation (70). A caveat to the approach of promoting dimer stability for SOD1-associated ALS mutants is that SOD1 dimer formation primarily occurs when monomers are already metal replete and disulfide oxidized: a species of SOD1, which is still highly stable even when containing ALS-associated mutations (71). Evidence points toward immature metal-depleted SOD1 being a precursor to the toxic forms of misfolded or aggregated SOD1 (4, 10, 18, 19) and, until recently, effective pharmacological chaperones targeting immature SOD1 were elusive. Ebselen is considered to have a potential dual effect on SOD1 maturation, facilitating disulfide formation, and increasing dimer affinity through binding at Cys111 (30), although it should be noted that *in vivo*, ebselen binding at Cys111 is unlikely because of the presence of reduced glutathione within cells. CuATSM is thought to facilitate the increased delivery of Cu to SOD1, increasing the pool of Cu-bound SOD1 in several SOD1 ALS animal models (31, 38, 40). Here, we considered that ebselen and CuATSM may be used in combination to promote proper SOD1 folding at two different points, Cu binding and disulfide formation in effect acting as a CCS mimetic.

Ebselen was found to be more effective at attenuating the inclusion formation and toxicity of WTL mutants when compared with SOD1^G85R^. Previous investigations have shown that SOD1 with Cu bound is less susceptible to disulfide reduction (59, 72, 73, 74). Since SOD1^A4V^ is a WTL mutant that retains Cu-binding capacity similar to that of WT, it would be expected that Cu-bound forms of SOD1^A4V^ would respond more favorably to ebselen-associated disulfide formation. Indeed, our data showed that combination treatment resulted in a greater level of enzymatically active SOD1^V148G^ as compared with either CuATSM or ebselen alone, suggesting a strong effect of polytherapy to stabilize even the most destabilizing ALS-associated mutants. In contrast, the pool of SOD1^G85R^ that binds Cu is relatively low (52, 74), meaning that the synergistic effect of Cu binding and disulfide formation that we observed for both SOD1^V148G^ and SOD1^A4V^ would not be expected to occur for SOD1^G85R^. In line with this, we saw no additive or synergistic effect of CuATSM in combination with ebselen against SOD1^G85R^ expression in our cell model.

Other pharmacological chaperones, such as those targeted against tryptophan-32 in SOD1 (28, 29, 75) or the molecular tweezer CLR01 (76), may result in more effective reduction of inclusion formation and toxicity in the case of SOD1 MBR mutants, such as SOD1^G85R^. Likewise, derivatives of ebselen may also prove more effective at reducing inclusion formation and toxicity in the case of SOD1 MBR mutants (42). Both ebselen and CuATSM are currently in human clinical trials. CuATSM is currently in clinical trials against ALS (NCT04082832 and NCT02870634), and ebselen is in clinical trials as a potential treatment for noise-induced hearing loss (77, 78) and is even being considered as a potential treatment for coronavirus disease 2019 (79). That these two compounds have available safety profiles is promising for their application as a polytherapy for ALS patients carrying SOD1 mutations. The effectiveness of CuATSM and ebselen for other forms of ALS is currently not well understood. CuATSM has been found to inhibit the paraquat-induced cytoplasmic localization of TAR DNA-binding protein 43 (TDP-43) into stress granules (80) and to prevent TDP-43 phosphorylation and fragmentation *in vivo* (81). Ebselen is yet to be examined against TDP-43-associated forms of ALS.

Collectively, we have shown here that a biophysical understanding of the folding pathway of a protein can be exploited to target it at key points to promote proper folding. However, the strategy presented here is by no means the only one that may be pursued against SOD1–fALS. Considering that the concept of proteostasis (protein homeostasis) incorporates protein synthesis, protein folding, protein trafficking, and protein degradation (82), there is potential to establish combination therapies against SOD1–fALS at multiple points. These therapies may upregulate the cellular chaperone networks (27), protein degradation pathways (83), or reduce SOD1 synthesis (84). For example, the most clinically promising therapeutics for SOD1-associated ALS are antisense oligonucleotides (84), which bind to and enhance the degradation of SOD1 mRNA, effectively reducing the concentration of SOD1 within a cell. Considering SOD1 deficiency may have negative effects in animals and humans (85), complete knockdown of SOD1 *via* antisense oligonucleotides may potentially lead to complications. A different strategy, to solely knocking down SOD1, may include partial knockdown of SOD1 paired with supplemental treatment with pharmacological chaperones such as CuATSM and ebselen to ensure that the SOD1 that is synthesized folds properly. Indeed, cancer researchers have made marked advances in establishing polytherapies as a highly effective method by which to treat cancer (55). Finally, future work against SOD1-associated ALS, and even other neurodegenerative diseases, should incorporate our growing knowledge of the root mechanisms and downstream effects into therapy design. We hypothesize that further improvement and expansion of the number of pharmacological chaperones that promote SOD1 folding will result in better outcomes in preclinical models and patients.

## Experimental procedures

### Plasmids for mammalian protein expression

Vectors for the expression of C-terminally EGFP-tagged SOD1 variants SOD1^WT^, SOD1^A4V^, SOD1^C6G^, SOD1^G37R^, SOD1^H46R^, SOD1^D90A^, SOD1^G93A^, SOD1^E100G^, SOD1^G127X^, and SOD1^V148G^ on a pEGFP-N1 backbone have been previously described (3, 41, 47). Plasmids for the expression of human SOD1^A^^4V^ from *Escherichia coli* have been described previously (30). Plasmids were heat transformed into subcloning efficiency chemically competent *E. coli* DH5α cells (Thermo Fisher Scientific) and purified using miniprep kits (Thermo Fisher Scientific) and maxiprep kits (Qiagen) as per the manufacturer's instructions.

### Mammalian tissue culture and transfection

NSC-34 (86) cells were cultured in Dulbecco's modified Eagle's medium-F12 (DMEM-F12) (Invitrogen), supplemented with 10% (v/v) heat-inactivated fetal bovine serum (Bovogen). In order to passage and plate NSC-34 cells, they were washed once with prewarmed DMEM-F12 and treated with 0.25% trypsin and 0.02% EDTA dissociation reagent (Invitrogen) to lift off the adherent cells. The cells were pelleted *via* centrifugation (300*g* for 5 min) and resuspended in prewarmed culture media. Following washing, plates were seeded at a confluency of 40% and cultured at 37 °C in a humidified incubator with 5% atmospheric CO_2_ for 24 h prior to transfection (∼70–80% confluent). Cells were transfected with plasmid DNA (0.5 μg per well of a 24-well plate, 2.5 μg per well of a 6-well plate) 24 h postplating using TransIT-X2 reagent (Mirus Bioscience) according to the manufacturer's instructions.

### Crystal violet assay for cell density

IC_50_ of various drugs on untransfected NSC-34 cells was determined *via* a crystal violet assay (0.5 g/l crystal violet, 1% methanol [v/v], 1× PBS) as previously described (87). NSC-34 cells were treated for 48 h with ebslen and CuATSM at concentrations ranging from 0 to 500 μM with a final concentration of 1% (v/v) (dimethyl sulfoxide [DMSO]; Sigma–Aldrich). The drug-treated NSC34 cells were then fixed *via* the addition of prewarmed 4% paraformaldehyde in 1× PBS and incubated for 30 min at room temperature prior to the addition of crystal violet solution. Crystal violet–stained cells were imaged using a Gel Doc XR+ gel imager (Bio-Rad). Glacial acetic acid (100 μl 33% [v/v]) was used to release the crystal violet stain back into solution for quantification of absorbance at 590 nm on a POLARstar plate reader (BMG Labtech). The resulting data were plotted *via* Prism (GraphPad Prism, version 5.00 or version 8.00; GraphPad Software, Inc) using a log (inhibitor) *versus* normalized response variable slope fit.

### Preparation of plates for fluorescence microscopy

NSC-34 cells were plated into 6-well culture plates at a confluency of 40% and incubated overnight at 37 °C in a humidified incubator with 5% atmospheric CO_2_. To overcome the effect of transfection efficiency differences within assays, cells were transfected as described previously and incubated for 5 h at 37 °C in a humidified incubator with 5% atmospheric CO_2_. Following incubation, cells were aspirated or lifted off with trypsin/EDTA dissociation reagent and replated into 96-well culture plates at a confluency of 30% in the presence or the absence of various compounds in a final volume of 100 μl and incubated for 48 h. Following incubation, cells in culture medium were fixed *via* addition of 100 μl prewarmed 4% paraformaldehyde in 1× PBS with a 30 min incubation. Following fixation, cells were permeabilized using 0.1% Triton X-100 in 1× PBS for 5 min, which was followed by a 5 min incubation in 1× PBS with a 1:5000 dilution of Hoescht 33342 (Life Technologies). Finally, cells were washed twice in 1× PBS before being immediately imaged or stored in the dark at 4 °C. Stored cells were imaged no later than 3 days after fixation.

### Fluorescence microscopy

A LionHeartFX automated microscope (Biotek Agilent) running Cytation software (Biotek Agilent, versions 3.04 and 3.08) was used for plate-based image acquisition. NSC-34 cells expressing SOD1 mutant and EGFP-tagged constructs were excited *via* illumination with a 465 nm light-emitting diode (LED), and the emission was filtered through a 469/525 nm bandpass filter cube. All images were taken using an UPLFLN PH 10× 0.3 numerical aperture objective (Olympus). Each well was imaged in a 4 × 4 or 5 × 5 tile scan. No image overlapping was used in order to avoid duplicating cell counts in later analysis stages. For imaging SOD1^WT^ transfected cells, the LED power was set to 1, and integration time was 20 ms to prevent the acquisition of saturated fluorescent signal from high expressing cells. Mutant SOD1-transfected cells were imaged using an LED power of 2 and an integration time of 25 ms, set to account for variation in fluorescent intensity and lower expression. A camera gain of 10 was consistent for both SOD1^WT^ and mutant samples. Metadata for individual wells were set to the following:

(WELL)_(IMAGE IN WELL)_(CHANNEL)_(IMAGE NUMBER) to generate a unique image identity such as B4_2_GFP_2 allowing for easy metadata assignment and data curation in image processing and analysis pipelines.

For live-cell imaging, similar settings were used as aforementioned with the LionHeartFX imager; however, cells were maintained in 5% atmospheric CO_2_ at 37 °C. Live-cell autofocusing was achieved using the standard brightfield autofocusing method that is performed by the LionHeartFX software.

### Image analysis

All images generated *via* automated microscopy underwent preprocessing quality control to omit out of focus images and to correct illumination variation in the datasets. Out of focus images were manually assessed by users and excluded from the dataset, whereas illumination variation within images was corrected using CellProfiler software modules *Correct Illumination Calculate* and *Correct Illumination Apply*. Briefly, a 500 pixel gaussian smoothing filter was used to generate illumination functions, which display the illumination variation within a set of images from one imaging session (multiple plates). The illumination function is then subtracted from the dataset to correct for variation across the image. After quality control processing, images were processed in CellProfiler to segment cells within the range of 17 to 50 pixel units and measure intensity, granularity, size/shape, intensity distribution, and texture. Accuracy of segmentation and thumbnail generation was assessed during the training of the machine learning algorithm. To determine accuracy, the user requested 100 cells of a particular “bin” and noted how many cells were incorrectly classified. This was repeated three times per number of cells the machine was trained on and the average accuracy noted. Once a reasonable accuracy was achieved (∼97%), all remaining cells were automatically scored *via* CellProfiler Analyst. Pipelines are available from the authors upon reasonable request.

Analysis of time-series data from live-cell imaging experiments was carried out similar to aforementioned with the focus on obtaining the number of GFP-transfected cells per well across the time series. To determine the relative survival of each treatment in the live-cell assays, analysis was carried out as previously described (3). Briefly, GFP-positive cells are counted in images and normalized to the starting number of cells. This value is then normalized to control values, which in this case are variants without drug treatment, to show comparative cell counts across time.

### Purified SOD1 free-thiol and homodimerization assays

Human recombinant SOD1^A^^4V^ was expressed in *E. coli* BL21 (DE3) and purified as described previously (42). The SOD1 intrasubunit disulfide bond was reduced with 40 mM DTT overnight at 4 °C followed by desalting into nitrogen purged 20 mM Tris–HCl and 150 mM NaCl with 5 mM reduced glutathione for size-exclusion chromatography homodimerization assays and without reduced glutathione for free-thiol assays. Compounds, including ebselen, were dissolved in DMSO and added to 20 μM SOD1-A4V at 20 or 100 μM concentration for homodimerization and free-thiol assays, respectively. To monitor recombinant SOD1-A4V disulfide formation *via* a free-thiol assay, the reaction was incubated at 20 °C for 1 h before addition of 400 μM 4-acetamido-4′-maleimidylstilbene-2,2′-disulfonic acid (AMS) and incubation at 37 °C for 90 min. Samples were then heated to 97 °C in nonreducing SDS sample buffer and then separated by SDS-PAGE using a 15% polyacrylamide gel. Homodimerization assay samples were incubated at 20 °C for 24 h and then 10 μl was loaded on an Agilent BioSEC Advance 300 Å, 4.6 × 300 mm size-exclusion chromatography column along with controls for disulfide-reduced and disulfide-intact forms of SOD1^A^^4V^ without ligands.

### Combenefit analysis for drug synergy

Following checkerboard drug treatment and determination of the percentage of transfected cells containing SOD1-EGFP inclusions, the data were processed for synergy analysis. First, checkerboard data were normalized as a percentage of the no drug control well prior to being input into spreadsheets in the format required by Combenefit software (59). Combenefit software was then performed on all datasets, and the HSA model outputs were examined for synergy values above 10.

### Immunoblotting of cell lysates

NSC-34 cells were cultured in 6-well plates, transfected, and treated with compounds similar to aforementioned methods. Importantly, following 6 h after addition of transfection complexes to cells, cells transfected with specific constructs (SOD1^WT^, SOD1^A^^4V^, or SOD1^G^^85R^) were lifted and mixed together and replated to ensure equal transfection per construct for each drug treatment. Following 48 h incubation in drugs (vehicle DMSO, 0.5 μM CuATSM, 20 μM ebselen, or a 0.5 μM CuATSM/20 μM ebselen combination), cells were washed with prewarmed (37 °C) serum-free DMEM/F12 once and incubated for 5 min in prewarmed 0.25% trypsin and 0.02% EDTA dissociation reagent. Once lifted, cells were harvested into microfuge tubes and pelleted at 300*g* for 5 min. Pellets were gently resuspended in prewarmed 1× PBS and spun again at 300*g* for 5 min. Supernatant was removed, and cell pellets were resuspended and lysed in 100 μl ice-cold 1× Tris-buffered saline (pH 7.4) with 1% TX-100 1 mg/ml *N*-ethylmaleimide supplemented with 1× Halt protease inhibitor (Thermo Fisher Scientific) to release soluble SOD1 from cells. Resuspensions were centrifuged at 20,000*g* for 20 min at 4 °C to pellet nuclei and insoluble material. Supernatants were carefully transferred to new microfuge tubes, and these samples were flash frozen with liquid nitrogen and stored at −80 °C prior to use.

Cell lysates were defrosted on ice and mixed 1:3 with either 4× nonreducing thiol-blocking SDS-PAGE sample buffer (200 mM Tris–HCl [pH 6.8], 8% SDS [w/v], 40% glycerol [v/v], 50 mM EDTA, 0.08% [w/v] bromophenol blue, and 40 mM *N*-ethylmaleimide) or 4× reducing SDS-PAGE sample buffer (200 mM Tris–HCl [pH 6.8], 8% SDS [w/v], 40% glycerol [v/v], 50 mM EDTA, 0.08% bromophenol blue [w/v], 4% β-mercaptoethanol [v/v]). Samples for SDS-PAGE were then heated to 95 °C for 5 min prior to being loaded onto 4 to 20% Criterion TGX Stain-Free gels (Bio-Rad). Gels were electrophoresed for 5 min at 100 V and then 1 h at 150 V. Following electrophoresis, total protein on the gel was quantified using a Criterion Stain Free Imager (Bio-Rad) prior to transferring for immunoblotting.

Proteins separated by SDS-PAGE were transferred onto methanol-activated Amersham Hybond 0.2 μm polyvinylidene fluoride membranes (GE Healthcare) at 100 V for 1 h at 4 °C using 1× transfer buffer (25 mM Tris-base, 20% methanol [v/v], and 192 mM glycine). Membranes were imaged with a stain-free imager post-transfer to confirm transfer and measure total protein. Following stain-free imaging, membranes were washed for 5 min in distilled water before being blocked in 1× Tris-buffered saline (pH 7.4) with 0.02% Tween-20 (v/v) (TBST) and 5% skim milk (w/v) at room temperature for 1 h. Following blocking, polyvinylidene fluoride membranes were incubated in polyclonal rabbit anti-GFP primary antibody (catalog no.: ab290; Abcam) diluted 1:5000 in TBST with 5% skim milk (w/v) at 4 °C overnight with tipping. Following overnight incubation in primary antibody, membranes were washed three times in TBST for 10 min per wash. Following washing, membranes were incubated in goat anti-rabbit horseradish peroxidase–conjugated secondary antibody (catalog no.:P0448; Dako) at a dilution of 1:5000 in TBST with 5% skim milk at room temperature for 1 h with tipping. Following secondary antibody incubation, membranes were washed three times in TBST prior to chemiluminescent detection of bands using SuperSignal West Pico Plus substrate (Thermo Fisher Scientific) and being imaged on a ChemiDoc MP Imaging System (Bio-Rad). Analysis and quantification was performed using ImageJ (version 1.53c, National Institutes of Health).

### In-gel zymography for SOD1 enzymatic activity

Cell lysates from transfected NSC-34 cells were generated as aforementioned, with the exception that lysis buffer was 100 mM Tris-base (pH 7.5) with 0.1% TX-100 (v/v) and protease inhibitor. Cell lysates were mixed 1:2 with 3× native-PAGE sample buffer (240 mM Tris–HCl [pH 6.8], 30% glycerol [v/v], 0.03% bromophenol blue [w/v]) and loaded into Tris–glycine native-PAGE gels (4.5% stacking gel [pH 8.8], 7.5% resolving gel [pH 8.8]). Samples were electrophoresed for 30 min at 60 V and then for 2.5 h at a constant voltage of 125 V at 4 °C. Following native-PAGE, EGFP, or TdTomato signal in the gel was detected using a ChemiDoc MP Imaging System (Bio-Rad). Gels were then subjected to zymography as described previously (64) and imaged using a GS-900 Calibrated Densitometer (Bio-Rad). Quantification of fluorescence signal and enzymatic activity was performed using ImageJ (version 1.53c) (88).

### Statistical analysis

All statistical analyses are described in the legends to the figures. All statistical analyses were performed using Prism software, version 5.00 or 8.00.

## Data availability

All data relating to this article are contained within the article.

## Supporting information

This article contains supporting information.

## Conflict of interest

L. M. reports that financial support was provided by 10.13039/100016887Motor Neurone Disease Research Australia. L. M. reports a relationship with University of Wollongong Illawarra Health and Medical Research Institute and Molecular Horizons Fluorescence Analysis Facility, both of which includes nonfinancial support. G. S. A. W. reports that financial support was provided by 10.13039/501100000406Motor Neurone Disease Association. All other authors declare that they have no conflicts of interest with the contents of this article.
