# Supplementary material for: A copper chaperone–mimetic polytherapy for SOD1-associated amyotrophic lateral sclerosis
Source: J Biol Chem. 2022 Jan 20;298(3):101612. doi: 10.1016/j.jbc.2022.101612 (PMC8885447; doi:10.1016/j.jbc.2022.101612)
Supplement: Supplement Table S1 [file mmc1.docx]

**Supplementary Table 1. List of ALS-associated SOD1 variants and their status as either ‘wild-type like’ or ‘metal-binding region’ mutants.**

| Wild-Type Like Mutants | Metal-Binding Regions Mutants | Truncation Mutants |
| --- | --- | --- |
| A4V  C6G  G37R  D90A  G90A  E100G  V148G | **H46R**  **G85R** | **G127X** |
